# The Action of Medicines on the System, Etc.

**Published:** 1853-08

**Authors:** 


*The Action of Medicines on the System, etc.* Being the Prize Essay to which the Medical Society of London awarded the Fothergillian Gold Medal for 1852. By FREDERICK WILLIAM HEADLAND, B. A., M. R. C. S., etc. Philadelphia: Lindsay & Blakiston. 1853. 8vo, pp. 560.

In this essay the author brings to bear on therapeutics the humoral views of pathology which prevail at the present time. The work is full of interest. Much of it is confessedly theoretical, but believing, as we do, that it is calculated to promote rightly directed inquiries and investigations, we unite most cordially with our contemporaries in commending it to our professional brethren.

The author adopts, as the basis of his essay, ten propositions, which we quote in order to give our readers a general idea of the objects and scope of the work:

PROP. 1. That the great majority of medicines must obtain entry into the blood or internal fluids of the body, before their action can be manifested.

PROP. 2. That the great majority of medicines are capable of solution in the gastric or intestinal secretions, and pass without material change, by a process of absorption, through the coats of the stomach and intestines, to enter the capillaries of the portal system of veins.

PROP. 3. That those medicines which are completely insoluble in water, and in the gastric and intestinal juices, cannot gain entrance into the circulation.

PROP. 4. That some remedial agents act locally on the mucous surface, either before absorption or without being absorbed at all. That they are chiefly as follows:
*a.* Irritant emetics.
*b.* Stomach anæsthetics.
*c.* Irritant cathartics.

PROP. 5. That medicine, when in the blood, must permeate the mass of the circulation, so far as may be required to reach the parts on which it tends to act.

That there are two possible exceptions to this rule:

*a.* The production of sensation or pain at a distant point.

*b.* The production of muscular contraction at a distant point.

Prop. 6. That while in the blood the medicine may undergo changes, which in some cases may, in others may not, affect its influence.

That these changes may be:

*a.* Of combination.

*b.* Of reconstruction.

*c.* Of decomposition.

Prop. 7. That a first class of medicines, called hæmatics, act while in the blood, which they infiuence. That their action is permanent.

1. That of these, some, called restoratives, act by supplying, or causing to be supplied, a material wanting; and may remain in the blood.

2. That others, called catalytics, act so as to counteract a morbid material or process; and must pass out of the body.

Prop. 8. That a second class of medicines, called neurotics, act by passing from the blood to the nerves or nerve-centers, which they influence. That they are transitory in action.

1. That of these some, called stimulants, act so as to exalt nervous force, in general or in particular.

2. That others, called narcotics, act so as first to exalt nervous force, and then to depress it; and have also a special influence on the intellectual part of the brain.

3. That others again, called sedatives, act so as to depress nervous force, in general or in particular.

Prop. 9. That a third class of medicines, called astringents, act by passing from the blood to muscular fiber, which they excite to contraction.

Prop. 10. That a fourth class of medicines, called eliminatives, act by passing out the blood through the glands, which they excite to the performance of their functions.

